# The Adsorption of Methylene Blue on Eco-Friendly Reduced Graphene Oxide

**DOI:** 10.3390/nano10040681

**Published:** 2020-04-04

**Authors:** Fabian Arias Arias, Marco Guevara, Talia Tene, Paola Angamarca, Raul Molina, Andrea Valarezo, Orlando Salguero, Cristian Vacacela Gomez, Melvin Arias, Lorenzo S. Caputi

**Affiliations:** 1 Grupo de Investigación de Materiales Avanzados, Facultad de Ciencias, Escuela Superior Politécnica de Chimborazo, Riobamba EC-060155, Ecuador; fabian.arias@espoch.edu.ec; 2 Faculty of Mechanical Engineering, Escuela Superior Politécnica de Chimborazo, Riobamba EC-060155, Ecuador; marco.guevara@espoch.edu.ec; 3 Grupo de Fisicoquímica de Materiales, Universidad Técnica Particular de Loja, Loja EC-110160, Ecuador; tbtene@utpl.edu.ec; 4 GraphenTech NL, Olympiaweg 28A, 3077AL Rotterdam, The Netherlands; paos19@hotmail.es (P.A.); rm@redecua.com (R.M.); 5 School of Chemical Sciences and Engineering, Yachay Tech University, Urcuquí EC-100119, Ecuador; andrea.valarezo@yachaytech.edu.ec; 6 CompNano, School of Physical Sciences and Nanotechnology, Yachay Tech University, Urcuquí EC-100119, Ecuador; orlando.salguero@yachaytech.edu.ec; 7 UNICARIBE Research Center, University of Calabria, I-87036 Rende (CS), Italy; melvin.arias@intec.edu.do; 8 Instituto Tecnológico de Santo Domingo, Área de Ciencias Básicas y Ambientales, Av. Los Próceres, Santo Domingo 10602, Dominican Republic; 9 Surface Nanoscience Group, Department of Physics, University of Calabria, Via P. Bucci, Cubo 33C, I-87036 Rende, Italy

**Keywords:** reduced graphene oxide, citric acid, methylene blue, adsorption

## Abstract

Recently, green-prepared oxidized graphenes have attracted huge interest in water purification and wastewater treatment. Herein, reduced graphene oxide (rGO) was prepared by a scalable and eco-friendly method, and its potential use for the removal of methylene blue (MB) from water systems, was explored. The present work includes the green protocol to produce rGO and respective spectroscopical and morphological characterizations, as well as several kinetics, isotherms, and thermodynamic analyses to successfully demonstrate the adsorption of MB. The pseudo-second-order model was appropriated to describe the adsorption kinetics of MB onto rGO, suggesting an equilibrium time of 30 min. Otherwise, the Langmuir model was more suitable to describe the adsorption isotherms, indicating a maximum adsorption capacity of 121.95 mg g^−1^ at 298 K. In addition, kinetics and thermodynamic analyses demonstrated that the adsorption of MB onto rGO can be treated as a mixed physisorption–chemisorption process described by H-bonding, electrostatic, and π − π interactions. These results show the potential of green-prepared rGO to remove cationic dyes from wastewater systems.

## 1. Introduction

Due to population growth and economic development, the demand for clean water is an urgent task [1]. However, the expansion of industrial (or agricultural) activities has caused the indiscriminate discharge of innumerable contaminants into aquatic systems such as organic pollutants, heavy metals, and dyes [2,3]. Unfortunately, current technologies are not able to remove all these contaminant agents to meet water-quality standards [4]. In this context, graphene-based nanomaterials appear as excellent platforms to treat water and wastewater [5,6]. With respect to conventional materials, graphene-based nanomaterials have attracted huge attention, particularly due to their large specific surface area and physical/chemical effects at the nanoscale [7]. Presently, several graphene-based or graphene-related [8,9,10] nanomaterials have been proposed as excellent water purification alternatives which promise to remove multiple contaminants [11].

Graphene is a one-atom-thick two-dimensional carbon material whose unique properties [12,13] (e.g., extraordinary electrical conductivity [14], high charge carrier mobility [15], high thermal conductivity [16], exceptional Young modulus [17], and tunable plasmons [18,19]) make it very attractive from the fundamental point of view and potential technological applications. For large-scale applications in environmental engineering, graphene can be prepared by liquid-phase exfoliation [20,21], shear-exfoliation [22,23] or oxidation/reduction [24,25]. The latter, particularly, is the most practical approach to prepare graphene oxide (GO) and reduced graphene oxide (rGO) with immediate applications in pollutants removal from aqueous media. Progress in this research area promises more efficient technologies that can replace or complement traditional ones [26].

Among industrial sectors, the textile industry is listed as one of the most polluting [27] due to the volumes of discharge and the composition of the contaminated effluent. Given the toxicological problems and environmental damage, the discharge of dyes is a matter of concern, even if the dyes are in very low concentrations (less than 1 ppm) [28,29]. The most common dye in the textile industry is methylene blue (MB) [30], which is a cationic dye and very soluble in water or alcohol. If MB is accidentally ingested, it can cause various poisoning problems and methemoglobinemia [31,32,33]. Hence, the removal of MB from aqueous media is a great challenge and urgently required.

To address this problem, several chemical, physical, and biological methods (e.g., flocculation precipitation [34], membrane separation [35], and electrolysis [36]) can be employed, but their efficiency are reduced since the dyes are slightly affected by light interaction, oxidation process or aerobic digestion [37]. Likewise, these traditional methods are complicated in operation, poor in decolorization efficiency, and generate sludge disposal problems. Thus, the adsorption methods are more attractive to treat contaminated water systems [38]. Furthermore, adsorption processes have been employed to test: (i) the interaction mechanism of phenolic compounds on GO and reduced GO [39], (ii) the efficiency of benzene/cyclohexane and toluene/cyclohexane binary mixtures into modified activated carbons to remove micropollutants [40,41], (iii) the restoration of a thallium (I)-contaminated aquifer by permeable adsorptive barriers [42], and (iv) the sorption of metaldehyde using granular activated carbon [43]. Keeping this in mind, the adsorption of dyes (or heavy metals [44]) has various advantages in the wide-scale treatment, for instance, low cost, environmental protection, and simple operation [45].

The adsorption of MB using pristine (or functionalized) oxidized graphene has been widely reported in the literature [38]. However, the adsorption mechanism of dyes on green-prepared rGO with potential scalability, is urgently required. The conventional oxidation processes (e.g., Hummer [46], Marcano [47,48,49], and Chen [50]), subsequent reduction steps, and final purification of the obtained material are a bottle neck when using GO and rGO for large-scale water treatment. Recently, we have demonstrated a scalable and eco-friendly protocol [51] to prepare oxidized graphene (GO and rGO) which is expected to be of immediate use in pollutants removal.

Although GO and rGO show similar adsorption efficiency [52], GO has a higher hydrophilic characteristic that could interfere with the removal of dyes from aqueous media. In fact, GO is highly dispersible in water which often leads to poor adsorption process. Additionally, GO–MB interaction is relatively weak, and chances always creep up for reversible adsorption of MB [53]. In this sense, the present work is focused on the removal of MB using our (pristine) eco-friendly rGO very recently reported. The equilibrium, kinetics, and thermodynamics of the adsorption process were investigated to study the adsorption mechanism of MB on rGO. In particular, the proposed method presents several advantages such as a complete and green synthesis of rGO, facile adsorption process, non-toxic gas evolution during the whole process [50], non-extra functionalization of rGO, and most importantly, a good adsorption efficiency compared to previous green [54] and non-green approaches.

## 2. Materials and Methods

### 2.1. Materials

**Figure 1:**
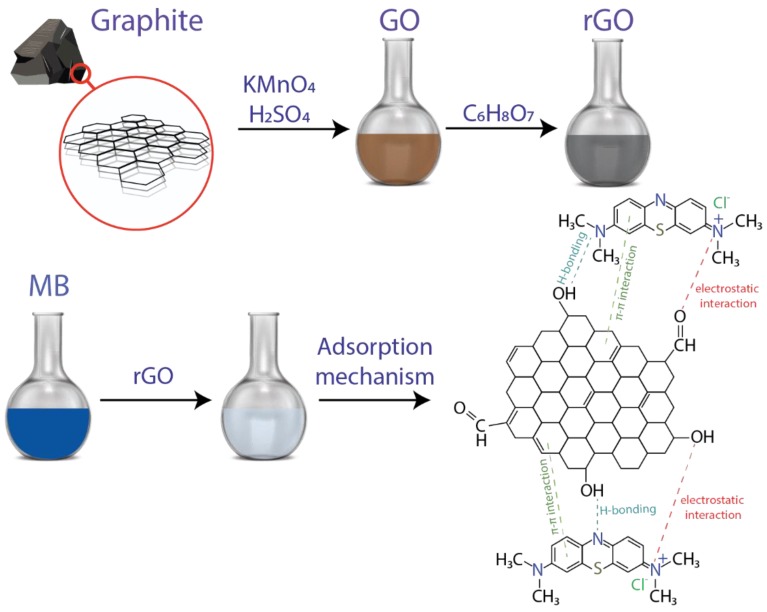
Schematic representation of the procedure and adsorption mechanism of methylene blue using reduced graphene oxide.

The materials used are reported in detail in [51]. Graphite powder (<150 μm, 99.99%), H_2_SO_4_ (sulfuric acid, ACS reagent, 95.0–98.0%), KMnO_4_ (potassium permanganate_,_ ACS reagent, ≥99.0%), HCl (citric acid, C_6_H_8_O_7_, ACS reagent, ≥99.5%), and MB (C_16_H_18_N_3_ClS, CAS Number 122965-43-9) were obtained from Sigma Aldrich (St. Louis, MO, USA). H_2_O_2_ (Hydrogen peroxide, 30%) and NaOH (Sodium hydroxide, 1310-73-2, 40.00 g/mol) were obtained from Merk (Kenilworth, NJ, USA). All chemicals were used as received. The process of synthesizing green-prepared (eco-friendly) rGO and the adsorption mechanics (hydrogen bonding, electrostatic and π−π interactions) of MB-rGO is shown in Figure 1.

### 2.2. Synthesis of GO and rGO

As commented above, GO and rGO were prepared by using our recently reported eco-friendly protocol [51]. The preparation procedure is briefly given as follows. Graphite powder (3.0 g) was added to H_2_SO_4_ (70 mL) under stirring in a water bath. Then, KMnO_4_ (9.0 g) was slowly added. The resulting mixture was agitated, and stirred by adding 150 mL distilled water at ~90 °C. In addition, 500 mL distilled water were employed, followed by a slow addition of 15 mL H_2_O_2_. The resulting solution was stirred up to change the color of the solution from dark brown to yellowish (Figure S1, Supplementary Materials). By centrifugation, the resulting suspension of graphite oxide was washed with HCl solution and distilled water up to adjust the pH ≈ 6. The obtained solid was dried to obtain graphite oxide powder.

For preparing rGO, 25 mg of graphite oxide powder was dispersed in 250 mL distilled water by sonication up to 0.5 h, employing an ultrasonic bath (Branson 2510 Ultrasonic Cleaner, Branson Ultrasonics Co., Brookfield, CT, USA). The resulting suspension was subjected to centrifugation for 0.5 h at 1000 rpm, to remove non-exfoliated particles. Then, 500 mg citric acid (CA) were gradually added to the centrifugated suspension under agitation. Different reaction times were tested from 0.5 to 1.5 h with the reduction temperature over 92 °C. The resultant precipitates were washed with distilled water by centrifugation for 0.5 h at 3000 rpm, and dried at 80 °C overnight to obtain rGO powder [51].

### 2.3. Characterization

The equipment used for the spectroscopical and morphological characterization of rGO, and sample preparation are reported in detail in [51]. The absorption spectra of GO and rGO, and spectra for absorbance studies were recorded using UV-Vis (ultraviolet-visible) spectroscopy (Thermo Scientific, Evolution 220, Waltham, MA, USA). The surface morphology of the obtained materials was taken out on a transmission electron microscope (TEM, JEM 1400 Plus, JEOL, Musashino, Akishima, Tokyo, Japan) operating at 80 kV, and a scanning electron microscope (SEM, JSM-IT100 InTouchScope, JEOL, Musashino, Akishima, Tokyo, Japan) with the accelerating voltage of 15 kV. Raman spectra were obtained using a Jasco NRS-500 (Easton, MD, USA) spectrometer with a 532 nm laser wavelength (0.3 mW, 100× objective).

### 2.4. Preparation of MB Solutions

MB powder was dissolved in ultra-pure water (18.3 MΩ cm) to get a stock solution of 1000 mg L^−1^. The standard working solutions were used in the tests through serial dilutions. By adding HCl and NaOH, the pH of the solutions was adjusted and measured using a pH meter (HI221 of Hanna Instruments, Woonsocket, RI, USA).

### 2.5. The Experiment of MB Adsorption on rGO

The adsorption investigations were carried out triplicate, including the synthesis of rGO, with a factorial experiment. To evaluate the adsorption kinetics and the effect of contact time (batch tests), 500 mg rGO were added into 250 mL of MB solution with a concentration of 100 mg L^−1^ in a 500 mL Erlenmeyer flask. The resulting mixture was agitated up to 8 h, considering three different temperatures (298, 313, 333 K). Similarly, adsorption isotherms were obtained from batch experiments adding 200 mg rGO in 50 mL of MB solutions with different concentrations in the range 10–100 mg L^−1^. The effect of pH was studied adding 200 mg rGO in 100 mL of MB solution with a concentration of 100 mg L^−1^. The removal of MB as function of rGO mass was determined by increasing the amount of rGO from 0.1 to 0.7 g in 50 mL of MB solution with a concentration of 100 mg L^−1^. Several aliquots were taken out from the solutions to be evaluated by UV-Vis measurements. The pH = 6.02 ± 0.07 was remained constant in all adsorption experiments. To test the effect of pH, the MB solution was fixed to the same initial pH value and adjusted by HCl (0.1 M) and NaOH (0.1 M) at room temperature, and immediately, rGO powder was gradually added.

The adsorption capacity ($q_{t}$, the amount of adsorbed MB per unit mass of adsorbent) was determined by [44]:

(1) $$q_{t}=\frac{\left(C_{0}-C_{t}\right)V}{W}$$

where $C_{0}$ and $C_{t}$ are the initial and MB ion concentrations (mg L^−1^) at time t, respectively. V represents the volume of the solution (L), and W is the adsorbent mass (g). At the adsorption equilibrium conditions, $C_{t}=C_{e}$ and $q_{t}=q_{e}$. $C_{e}$ is the equilibrium concentration, and $q_{e}$ denotes the equilibrium adsorption capacity. The removal efficiency (RE%) of rGO can be calculated as follow [44]:

(2) $$RE\%=\frac{\left(C_{o}-C_{e}\right)}{C_{o}}\times 100$$

## 3. Results and Discussions

Here, we quickly describe the transformation of GO into rGO. A detailed characterization of the eco-friendly prepared GO and rGO was reported in [51]. In particular, the guideline protocol, very recently reported, can be implemented for the large-scale production of oxidized graphenes through a reliable method, short-sonication time, and simple washing steps. Keeping this in mind, the main intent of the present work is to propose a general, green approach for the removal of MB using rGO. Thus, we have successfully demonstrated that CA (without an extra functionalization or treatment) is a reasonable reducing agent compared to conventional reducing chemicals, e.g., hydrazine hydrate, hydroquinone, sodium borohydride, and hydrogen sulfide.

### 3.1. Transformation of GO into rGO

**Figure 2:**
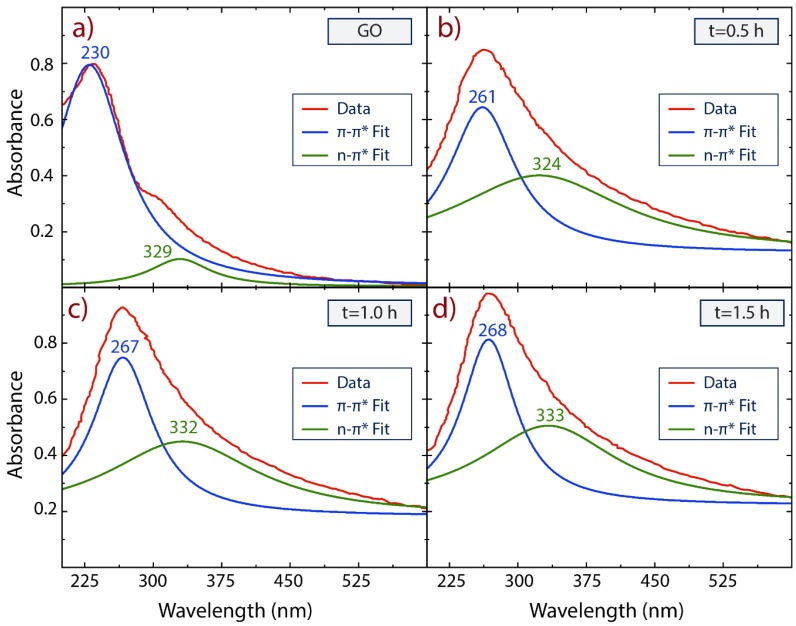
UV-Vis spectra recorded in aqueous solution at 0.1 mg/mL of (**a**) GO and (**b**–**d**) rGO as a function of the reduction time by using citric acid. The characteristic absorbance peak of GO and rGO were fitted with two Lorentzian functions.

The reduction of GO into rGO was first confirmed by UV-Vis absorption spectroscopy and the observed results are shown in Figure 2 and Figure S1. To explore the effect of the reduction time, we have fitted the characteristic absorbance peaks of GO and rGO with two Lorentzian functions. GO (Figure 2a) exhibits a main adsorption peak at 230 nm (blue line fit) and a less intense peak at 329 nm (green line fit), which are attributed to the π−π * transitions of C−C and n−π * transitions of C=O bonds, respectively [51,55]. Interesting enough, the absorbance peak of GO observed at 230 nm gradually redshifts to 268 nm and the absorbance intensity (>250 nm) increases with the reaction time (Figure S1), suggesting that the electronic conjugation of aromatic structure is restored upon CA reduction. This result shows that the electronic conjugation level of graphene can be chemically controlled, offering possibilities for tailoring the optical and electrical properties of rGO [55], with possible uses in photocatalytic applications.

While the electronic conjugation is recovered and rGO spectra seem to be featureless in the visible region as expected for graphene, the n−π * transitions (shoulder peak) observed in GO are relatively affected by reduction, even if the reduction time is increased (Figure 2b–d). The n−π * peak (green line fit) slightly redshifts from 329 nm to 333 nm; however, the related values of the full-width-of-half-maximum (FWHM) dramatically increase from 81.1 ± 8.3 nm to 180.2 ± 13.0 nm. These results evidence the presence of oxygen-containing functional groups (i.e., hydroxyl, epoxide, carbonyl, and carboxyl dominant groups [56]) attached to the basal plane or at the edges of the graphene structure [57] even when its surface area begins to recover. This chemical feature is a crucial result from the point of view of the removal of organics pollutants, because three main characteristics are needed in graphene to treat dyes, such as MB: large π interactions, electronegativity, and large specific surface area [7].

In our previous work, we have demonstrated by EDS (energy-dispersive X-ray spectroscopy) measurements that increasing the reduction time (>2 h), a partial oxidation is produced which might be attributed to a partial oxygen functionalization from water molecules because the reduction procedure was carried out at 95 °C [51]. We then focus on the reduction at 0.5 h to optimize the production process of rGO, avoiding a probable instable reduction, and preserving, particularly, a reasonable electronegativity.

**Figure 3:**
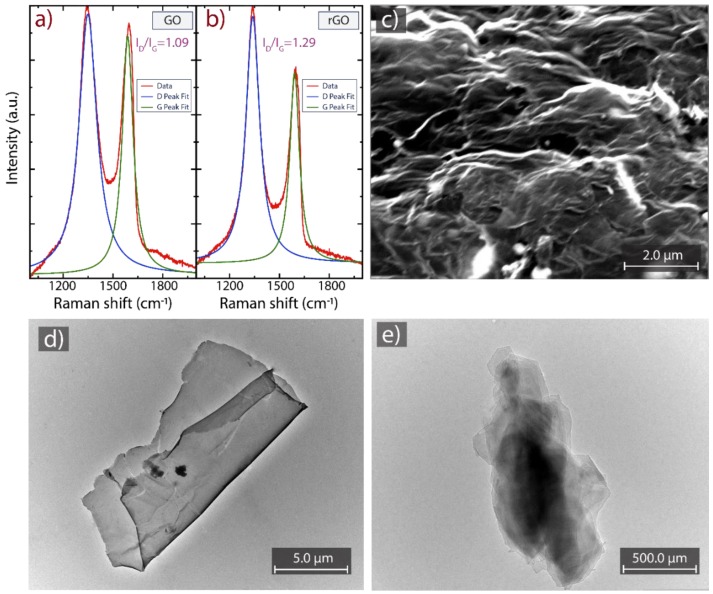
(**a**,**b**) Raman spectra recorded using 532 nm laser excitation. The intensity was normalized by the D peak. (**c**) SEM morphology of rGO. TEM images of (**d**) GO and (**e**) rGO.

Raman spectroscopy has been the major experimental technique to study the bonding nature of various carbon materials [58]. To further characterize the reduction efficiency, Raman analyses were performed on GO and compared with rGO as shown in Figure 3a,b, respectively. As is typical for oxidized graphenes, two prominent peaks are detected, i.e., the D peak at ~1349 cm^−1^ (~1340 cm^−1^) and the G peak at ~1588 cm^−1^ (~1592 cm^−1^). Other less intense peaks also have been detected (and discussed in [51]) in the range from 2700 cm^−1^ to ~3000 cm^−1^.

The D peak is due to the breathing mode of aromatic carbon rings, which is Raman inactive in perfect graphene and is activated by structural defects. The G peak, on the other hand, is due to the C–C stretching mode in sp^2^ hybridized carbon, and the *I_D_*/*I_G_* peak intensity ratio can be used as an indicator of the density of structural defects in graphenic materials [58,59,60]. However, a very high density of defects can strongly affect the density and structure of carbon rings. In such cases, the D intensity has a reverse meaning, and the *I_D_*/*I_G_* ratio must be interpreted with caution [61]. In our case, we observe an increase of the *I_D_*/*I_G_* ratio after reduction, accompanied by a reduction of the FWHM. In fact, the FWHM values change from 130.12 ± 1.12 cm^−1^ (D peak fitted, blue line) and 79.73 ± 0.92 cm^−1^ (G peak fitted, green line) to 100.21 ± 0.88 cm^−1^ (D peak fitted, blue line) and 69.57 ± 0.91 cm^−1^ (G peak fitted, green line) for GO and rGO, respectively. A reduction of the FWHM can be interpreted as due to an increased level of structural order of the system. In fact, in “*disordered*” graphenic systems, the width of the D peak results higher with respect to graphene, due to the contributions of breathing modes attributable to quasi-hexagons in carbon clusters [62]. Therefore, we interpret the increase of the *I_D_*/*I_G_* ratio and the decrease of the FWHM values as a reduction-induced recover of the density and structural quality of carbon aromatic rings. Very similar Raman results have been reported by Krishnamoorthy et al. in their investigation of the reduction of GO by D-galactose [58].

SEM and TEM micrographs of GO and rGO are shown in Figure 3c–e. The surface morphology of rGO (Figure 3c) exhibits randomly organized layers. The observed highly distorted surface can prevent face-to-face stacking of the graphene layers by the formation of mesopores and macropores as demonstrated in [51]. The obtained layers in rGO seem to be free of impurities. The latter is confirmed by TEM analysis. Figure 3d,e show representative TEM images of GO and rGO, respectively. A transparent and thin nanosheet with some wrinkles and folds on the surface and edges is observed for GO. In the case of rGO, well-defined graphene nanosheets were obtained after reduction, suggesting that rGO did not undergo severe basal-plane disruption compared to GO. However, the observed dark region can be attributed to the reduction of oxygen-containing functional groups which allows the partial re-stacking of graphene nanosheets. This outcome is under study by reducing the eco-friendly prepared GO with different temperatures, reducing agents, and reaction times.

### 3.2. Adsorption Kinetics

**Figure 4:**
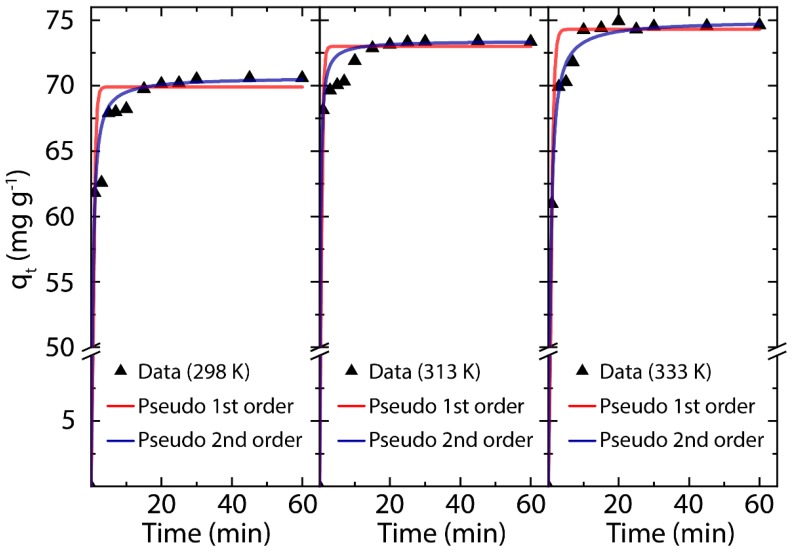
Adsorption kinetics of MB on rGO as a function of contact time (up to 60 min) and different temperatures (298, 313, 333 K).

We now move to the focus of the article, the adsorption mechanism of MB onto eco-friendly prepared rGO (Figure 1). The adsorption kinetic curves of MB onto rGO (and the influence of the contact time up to 60 min), considering three different temperatures (298, 313, and 333 K), are depicted in Figure 4. It can be seen that the adsorption equilibrium time was reached after 30 min, which is optically demonstrated in Figure S2. It should be noted that MB can be degraded by solar radiation; however, Eskizeybek and coworkers have demonstrated that MB can be degraded around 2% after five hours exposure to natural or UV light irradiation [38]. Hence, we assume that the effect of solar radiation is negligible in the present study.

The adsorption kinetics parameters were determined using the pseudo-first and pseudo-second-order models. The pseudo-first model assumes that the rate of change of the adsorption capacity is proportional to the concentration of available active sites per unit mass of adsorbent material [44,63,64], and can be calculated using the following expressions:

(3) $$\frac{dq_{t}}{dt}=k_{1}\left(q_{e}-q_{t}\right)$$

(4) $$\log\;\left(q_{e}-q_{t}\right)=\log q_{e}-\frac{k_{1}}{2.303}t$$

where $q_{t}$ is the adsorption capacity at a specific time (t) and $q_{e}$ represents the equilibrium adsorption capacity. In contrast, the pseudo-second model assumes that the rate of change of the concentration of occupied active sites per unit mass of the adsorbent material, is proportional to the square of the concentration of free active sites per unit mass of sorbent [44,65] and can be described as:

(5) $$\frac{dq_{t}}{dt}=k_{2}{\left(q_{e}-q_{t}\right)}^{2}$$

(6) $$\frac{t}{q_{t}}=\frac{1}{k_{2}q_{e}^{2}}+\frac{1}{q_{e}}t$$

where $q_{e}$ and $q_{t}$ are the concentrations of MB per unit mass at equilibrium and time (t), respectively. The equilibrium adsorption time obtained in the kinetic experiment will be used as reference time in subsequent adsorption processes. The parameters of the adsorption kinetics of MB on rGO are summarized in Table 1. From the pseudo-first and pseudo-second-order model, the calculated *q_e(cal)_* values are very close to the experimental ones. Interestingly, the experimental *q_e(exp)_* and calculated *q_e(cal)_* values slightly increase as a direct effect of temperature. By comparison of the SSE (sum square error) values in the kinetic models used, the adsorption kinetics of rGO is more in line with the pseudo-second-order model (as demonstrated in Figure 4, blue line fits), suggesting that the adsorption process of MB onto rGO could be controlled by chemisorption [44,65] independently of temperature. This asseveration will be discussed in detail in the adsorption thermodynamics section.

### 3.3. Particle Diffusion

**Figure 5:**
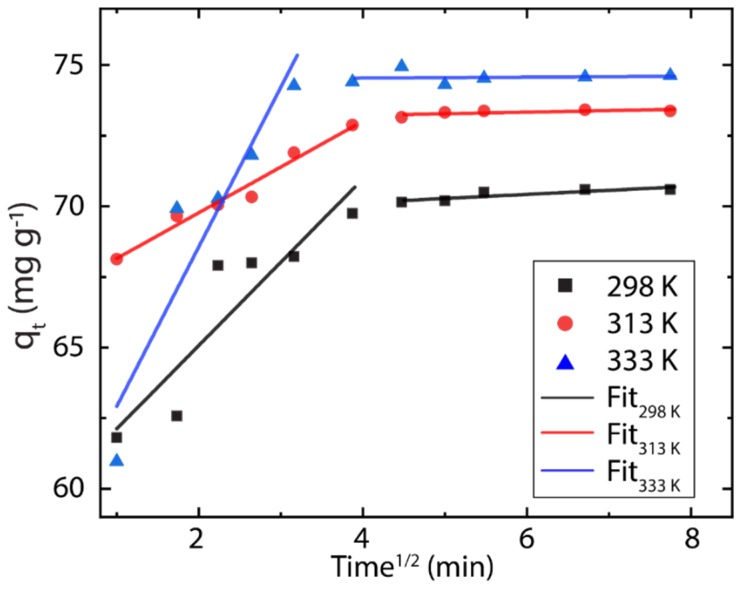
Intraparticle diffusion (IPD) plot showing two regions of linearity (*C_0_* = 100 mg L^−1^, *V* = 250 mL, *W* = 500 mg) at different temperatures.

The adsorption mechanism of dyes on solid porous surfaces mostly involves several steps characterized by different rates, in which the solute diffuses into the adsorbent material [44]. Indeed, the adsorption process of MB onto rGO can be studied by the intraparticle diffusion (IPD) model proposed by Weber et al. [66], and fitted as follow:

(7) $$q_{t}=k_{p}t^{1/2}+C$$

where $k_{p}$ is the rate constant of the IPD and C, the intercept at $q_{t}=0$, is a constant for the process. Thus, the linearized plot of $q_{t}$ (mg g^−1^) as function of $t^{1/2}$ (min) based on IPD model is shown in Figure 5. For large-time adsorption processes, three regions commonly are identified, corresponding to different diffusion steps: (i) the initial (most rapid one) is related to the external surface adsorption in which the adsorbed species move from the solution to the absorbent surface, (ii) the second region is related to the gradual diffusion of the solute into the pores of the absorbent material, and (iii) the final equilibrium process involves a very slow diffusion of the adsorbent species from larger pores to smaller ones [44].

To scrutinize the above-mentioned regions on the MB-rGO system, first, the initial adsorption factor (*R_i_*) should be calculated by the following expression:

(8) $$R_{i}=\frac{q_{ref}-C}{q_{ref}}$$

where $q_{ref}$ is the adsorption capacity at the longest time and C represents the boundary layer thickness [67]. The parameters of the IPD model are summarized in Table 2.

In MB-rGO system, the first and second regions are observed (independently of temperature); however, the third region (related to very slow diffusions) is obviously absent because the absorption of MB is reached at ~30 min. As expected, the calculated *R_i_* values are less than 0.5 indicating that the adsorption kinetics has a strong extent of surface adsorption [67], which means that most of the adsorption of MB occurs on the surface of rGO. The latter confirms that the large specific surface area of graphene begins to be recovered after the reduction process.

The adsorption isotherm analysis was carried out to study the interaction between MB molecule and rGO, with a span of 30 min. The latter is the equilibrium time obtained from the adsorption kinetics data. The adsorption isotherm was modeled using the Freundlich and Langmuir models, which can be represented mathematically as follow:

(9) $$\log q_{e}=\log K_{F}+\frac{1}{n}\log C_{e}$$

(10) $$q_{e}=\frac{q_{m}K_{L}C_{e}}{1+K_{L}\;C_{e}}\;$$

**Figure 6:**
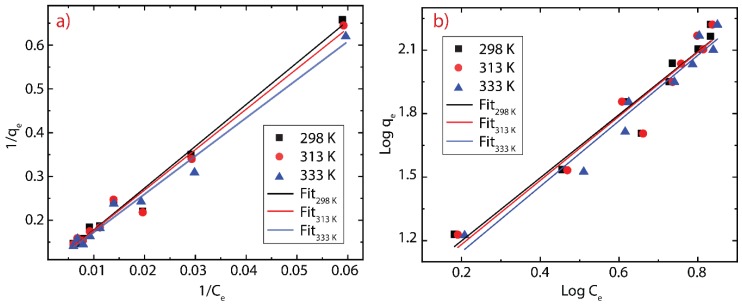
Adsorption isotherm of MB on rGO at different temperatures. (**a**) Langmuir model and (**b**) Freundlich model.

In the Freundlich model, $K_{F}$ is the adsorption capacity (mg L^−1^), n is the heterogeneity of rGO. In the Langmuir model, $q_{m}$ represents the maximum adsorption capacity, $K_{L}$ is the Langmuir constant. The resulting analysis is presented in Figure 6 and the parameters of both models are summarized in Table 3.

Based on the *R^2^* obtained, it can be seen the Langmuir model is more in line with data, compared to the Freundlich model. Furthermore, n values indicate that the adsorbent heterogeneity is minimal and tends to be homogeneous [54]. This result suggests a uniformity of the adsorption surface. The affinity of the adsorption between MB-rGO also can be determined by the *K_L_* parameter, where the value is very small (>0.1); which supports the results of data n from the Freundlich model [54]. Although the temperature seems to play a minor role on the adsorption of MB onto rGO (Figure S3), we clearly evidence that by increasing the temperature (from 298–333 K) the maximum adsorption capacity decreases (from 121.95–107.53 mg g^−1^). The temperature is an important parameter to be considered if rGO is used for industrial applications on dyes (or pollutants) removal. It is worth noting that the maximum adsorption capacity of green-prepared rGO is lower compared to that of [54]. This result may be attributed to the low $C_{e}$ values currently scrutinized (Figure S3). However, the *q_m_* = 121.95 mg g^−1^ is higher than previous studies (Table 4), suggesting that our green-prepared rGO is an excellent option for conventional materials. Although activated carbon seems to be more profitable to be used for the removal of MB [68] (Table 4), its adsorption equilibrium time (150 min) is higher compared to the present study (30 min), most importantly, rGO has a wide range of applications from composites to high frequency devices [21].

### 3.4. pH Effect

**Figure 7:**
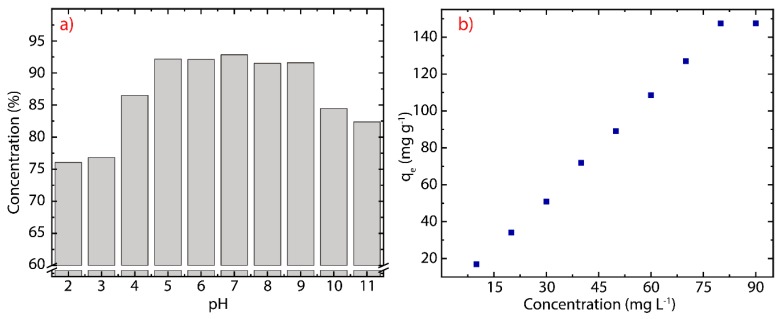
(**a**) Adsorption of MB on rGO as function of the pH (*C_0_* = 100 mg L^−1^, *W* = 200 mg, *T* = 298) and (**b**) effect of the initial concentration on the adsorption process (*C_0_* = 10–90 mg L^−1^, *W* = 200 mg, *V* = 50 mL).

To investigate the effect of pH on the adsorption capacity of the rGO nanosheets (Figure 3e), the experiments were carried out at ten different pH values (from 2 to 11) at 298 K, and the results are shown in Figure 7a. The adsorption increases with pH starting from removal percentages of about 76% at pH=3 up to about 93% at pH=7. The adsorption capacity, particularly, remains relatively constant for 5≤pH≤9. In contrast, the adsorption capacity decreases up to about 83% for pH ≥10. To give a simple description of this phenomenon, the effect of pH can be divided in three regions, and explained by electrostatic interactions: (i) the first region (2≤pH≤4) is rich in cations, typical of the acidic medium, which are adsorbed together with the cationic dye molecules, (ii) the second region (5≤pH≤9) is practically neutral because the number of cations decrease as the pH increases, consequently, only cationic dye molecules interact with the surface of rGO, and (iii) the third region (10≤pH≤11) has excess oxygens, i.e., ${\mathrm{OH}}^{-}$ ions, which interact with the cationic dye molecules, remaining suspended in the solution. These outcomes call for further experiments to verify the adsorption capacity of green-prepared rGO [51,54] by testing non-cationic dyes. It is important to stress that previous studies have limited the pH values up to 9 (e.g., [52,77]), and an explanation of the effect of pH remained ambiguous. Interestingly, Figure 7b demonstrates that the adsorption capacity of rGO increases as the MB concentration increases, suggesting that rGO offers many active sites for the adsorption of MB molecules. This result confirms an extensive surface area of rGO recovered after reduction. The latter observation could be useful to characterize the specific surface area of rGO subjected to different chemical or thermal reduction treatments.

### 3.5. Adsorption Thermodynamics

**Figure 8:**
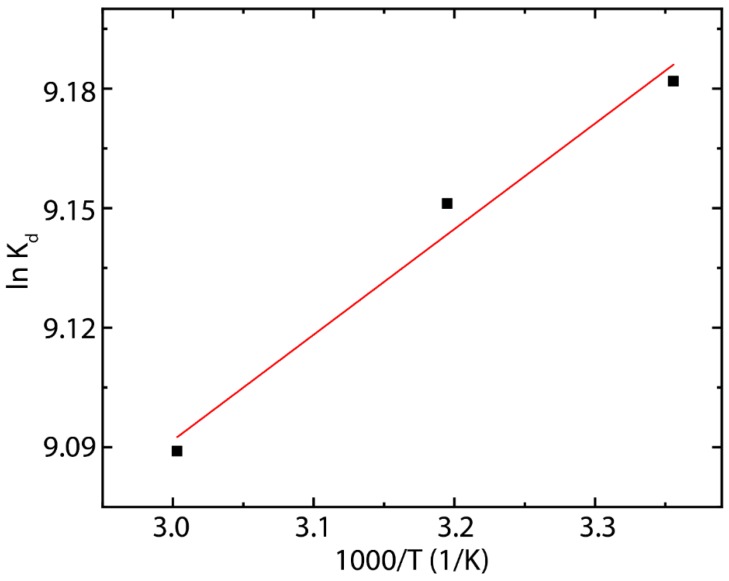
Van’t Hoff plot for the adsorption of MB on rGO.

To get information about the energy changes due to adsorption process of MB onto rGO nanosheets, thermodynamic parameters, i.e., the standard Gibbs free energy change ($\Delta G^{0}$), enthalpy change ($\Delta H^{0}$), and entropy change ($\Delta S^{0}$) were determined by using the following equations [78]:

(11) $$K_{d}=\frac{q_{e}}{C_{e}}$$

(12) $$ln\;K_{d}=\frac{\Delta S^{0}}{R}-\frac{\Delta H^{0}}{RT}$$

(13) $$\Delta G^{0}=-RT\;lnK_{d}\;$$

where $K_{d}$ is the distribution coefficient, T is the temperature, R is the gas constant (R=8.314 J mol−1 K−1), and $\Delta H^{0}$ and $\Delta S^{0}$ are calculated from the slope and intercept of Van’t Hoff plot of ln $K_{d}$ as function of $T^{-1}$ (Figure 8). The calculated thermodynamic parameters are given in Table 5.

The observed negative *ΔG^0^* values (at different temperatures) indicate the spontaneous nature of the adsorption of MB onto rGO nanosheets, which is also reflected from faster equilibrium time of the present MB-rGO system compared to previous MB-rGO and MB-GO systems [52]. The *ΔG^0^* values in the range from −22.75 to −25.16 kJ mol^−1^ indicates that the adsorption process of MB onto green-prepared rGO can be treated as a mixed physisorption–chemisorption process. Indeed, the adsorption process is assigned to physisorption in nature when the *ΔG^0^* value is in the range from 0 to −20 kJ mol^−1^, while *ΔG^0^* values in the range from −80 to −400 kJ mol^−1^ suggest a chemisorption process [52]. The partition between these two categories is vague [79]. Then, based on isotherm models it was seen that in this (MB-rGO) process both monolayer adsorption (chemisorption) and multilayer adsorption (physisorption) can occur.

Interestingly, as the temperature increases, the *ΔG^0^* value also increases, indicating a stronger interaction between MB and rGO. Nevertheless, negative $\Delta H^{0}$ values represent the exothermic nature of the adsorption of MB by rGO adsorbent, say, a negative enthalpy (−2.20 kJ mol^−1^) implies that temperature increase had negative impact on the adsorption of MB, resulting in higher adsorption at lower temperatures. Negative $\Delta H^{0}$ values, particularly, indicate that high temperature during adsorption process causes low adsorption efficiency [79]. The positive value of $\Delta S^{0}=0.069$ confirms the good affinity of MB toward the rGO nanosheets, and the increased randomness at the rGO-water interface during the adsorption process [52]. In addition, this result also supports that some structural changes occur on rGO nanostructure.

The adsorption isotherms and adsorption thermodynamics allow a conclusion that the adsorption mechanism of MB onto eco-friendly prepared rGO is governed by H-bonding, electrostatic and π−π interactions as seen in Figure 1. Accordingly, the adsorption of MB on the surface of rGO could go up to 121.95 mg g^−1^. This result can be improved by controlling the reduction process with other green-reducing agents such as reported in [54].

## 4. Conclusions

In summary, the adsorption mechanism of MB onto eco-friendly prepared rGO has been investigated. The rGO nanosheets are better adsorbents compared to previously reported (pristine or functionalized) rGO nanosheets for the removal of MB from water systems. The pH and temperature affect the adsorption efficiency of rGO nanosheets. The adsorption of MB onto rGO increases with increase to the pH up to 9, whereas it decreases with increase to pH ≥10. The maximum adsorption capacity of rGO was found to be 121.95 mg g^−1^. The adsorption of MB onto rGO followed a pseudo-second-order model. For the isotherm process, the MB-rGO system is more in line with the Langmuir model. However, the adsorption kinetics and adsorption thermodynamics demonstrated a mixed physisorption–chemisorption process. The adsorption of MB was an exothermic process. The present study proposes pristine rGO as potential adsorbent and green material to treat water or wastewater, most importantly, with a potential scalability on an industrial-scale application of graphene-based nanomaterials.

## Figures and Tables

**Table 1:** Parameters of the pseudo-first-order and pseudo-second-order kinetic model, considering different temperatures.

	Temperature		
Parameters	298 K	313 K	333 K
Experimental			
*q_e(exp)_* (mg g^−1^)	68.21	71.78	72.24
Pseudo-first-order model			
*q_e(cal)_* (mg g^−1^)	69.82	72.90	74.36
*k*_1_ (min^−1^)	2.166	2.727	1.714
SSE	13.38	27.07	7.75
*R*^2^	0.997	0.994	0.998
RMSE	1.157	1.645	0.884
Pseudo-second-order model			
*q_e(cal)_* (mg g^−1^)	70.72	73.43	75.09
*k*_2_ (g mg^−1^ min^−1^)	0.075	0.175	0.056
SSE	5.239	2.552	4.497
*R*^2^	0.999	0.999	0.999
RMSE	0.724	0.505	0.671

**Table 2:** Parameters of the intraparticle diffusion (IPD) model for the MB adsorption on rGO, considering different temperatures.

	Temperature		
Parameters	298 K	313 K	333 K
IPD model			
*R_i_*	0.132	0.073	0.207
*K_p_* (mg g^−^^1^ min^−^^1/2^)	2.95 ± 0.67	1.62 ± 0.14	5.65 ± 1.26
*C* (mg g^−^^1^)	59.17 ± 1.75	66.53 ± 0.37	57.27 ± 2.86
*R*^2^	0.829	0.971	0.871

**Table 3:** Parameters of Langmuir and Freundlich isotherm adsorption model, considering different temperatures.

T (K)	Langmuir Model			Freundlich Model		
	*K_L_* (L g^−1^)	*q_m(cal)_* (mg g^−^^1^)	*R* ^2^	*K_F_* (mg^(1^^−^*^n^*^)^ g^−^^1^ L^1/*n*^)	*n*	*R* ^2^
298	0.079 ± 0.0003	121.95 ± 4.11	0.982	7.956 ± 0.084	0.671 ± 5.5 × 10^−5^	0.945
313	0.081 ± 0.0004	116.28 ± 5.71	0.980	7.568 ± 0.101	0.661 ± 0.006	0.936
333	0.082 ± 0.0002	107.53 ± 2.85	0.984	6.869 ± 0.075	0.646 ± 0.004	0.955

**Table 4:** Comparative adsorption capacity of several adsorbents for the removal of MB.

Adsorbents	Adsorption Capacity (mg g−1)	References
Graphene/SrAl_2_O_3_:Bi^3+^	42.92	[69]
ß-cyclodextrin/MGO	93.97	[70]
g-C_3_N_4_ (Urea)	2.51	[71]
TiO_2_/Na-g-C_3_N_4_	1.80	[72]
Magnetic carboxyl functional nanoporous polymer	57.74	[73]
CeO_2_	4.37	[74]
Fe_3_O_4_—rGO-TiO_2_	1.67	[75]
Ag-Fe_3_O_4_—polydopamine	45.00	[76]
Citrus hystrix—rGO	276.06	[54]
HT—activated carbons	714–847	[68]
Eco-friendly rGO	121.95	Present work

**Table 5:** Thermodynamic parameters for MB adsorption on rGO at different temperatures.

*T* (K)	*ΔG*^0^ (kJ mol^−1^)	*ΔH*^0^ (kJ mol^−1^)	*ΔS*^0^ (kJ mol^−1^ K^−1^)
298	−22.75	−2.20 ± 0.04	0.069 ± 1.46 × 10^−5^
313	−23.81		
333	−25.16		

## Supplementary Materials

The following are available online at https://www.mdpi.com/2079-4991/10/4/681/s1, Figure S1: UV-Vis spectra recorded in aqueous solutions at 0.1 mg/mL of rGO considering different reduction times (0.5 h, 1.0 h and 1.5 h). The intensity was normalized by the predominant peak; Figure S2: Optical image for adsorption of MB onto rGO at *T* = 298 K. The MB-rGO suspension was centrifugated at 3000 rpm for 5 min after 30 min of contact time; Figure S3: Experimental adsorption isotherms for the removal of MB at different temperatures.
