# Supplementary material for: The Adsorption of Methylene Blue on Eco-Friendly Reduced Graphene Oxide
Source: Nanomaterials (Basel). 2020 Apr 4;10(4):681. doi: 10.3390/nano10040681 (PMC7221676; doi:10.3390/nano10040681)
Supplement: Supplementary file 1 [file nanomaterials-10-00681-s001.pdf]

nanomaterials

Supplementary Materials: The Adsorption of Methylene Blue on Eco-Friendly Reduced Graphene Oxide

Fabian Arias Arias [1], Marco Guevara [2], Talia Tene [3], Paola Angamarca [4], Raul Molina [4], Andrea Valarezo [5], Orlando Salguero [6], Cristian Vacacela Gomez [6,7,*], Melvin Arias [7,8] and Lorenzo S. Caputi [7,9,*]

1 Grupo de Investigación de Materiales Avanzados, Facultad de Ciencias, Escuela Superior Politécnica de Chimborazo, Riobamba EC-060155, Ecuador; fabian.arias@espoch.edu.ec
2 Faculty of Mechanical Engineering, Escuela Superior Politécnica de Chimborazo, Riobamba EC-060155, Ecuador; marco.guevara@espoch.edu.ec
3 Grupo de Fisicoquímica de Materiales, Universidad Técnica Particular de Loja, Loja EC-110160, Ecuador; tbtene@utpl.edu.ec
4 GraphenTech NL, Olympiaweg 28A, 3077AL Rotterdam, The Netherlands; paos19@hotmail.es (P.A.); rm@redecua.com (R.M.)
5 School of Chemical Sciences and Engineering, Yachay Tech University, Urcuquí EC-100119, Ecuador; andrea.valarezo@yachaytech.edu.ec
6 CompNano, School of Physical Sciences and Nanotechnology, Yachay Tech University, Urcuquí EC-100119, Ecuador; orlando.salguero@yachaytech.edu.ec
7 UNICARIBE Research Center, University of Calabria, I-87036 Rende (CS), Italy; melvin.arias@intec.edu.do
8 Instituto Tecnológico de Santo Domingo, Área de Ciencias Básicas y Ambientales, Av. Los Próceres, Santo Domingo 10602, Dominican Republic
9 Surface Nanoscience Group, Department of Physics, University of Calabria, Via P. Bucci, Cubo 33C, I-87036 Rende, Italy
* Correspondence: cvacacela@yachaytech.edu.ec (C.V.G.); lorenzo.caputi@fis.unical.it (L.S.C.)

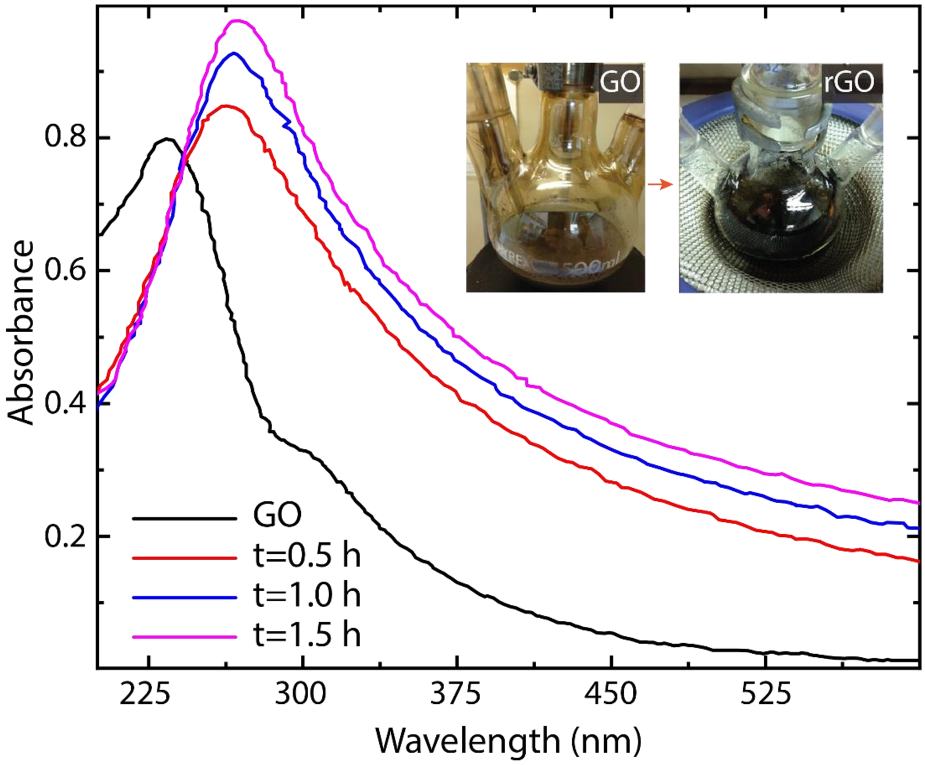

Figure S1. UV-Vis spectra recorded in aqueous solutions at 0.1 mg/mL of rGO considering different reduction times (0.5 h, 1.0 h and 1.5 h). The intensity was normalized by the predominant peak.

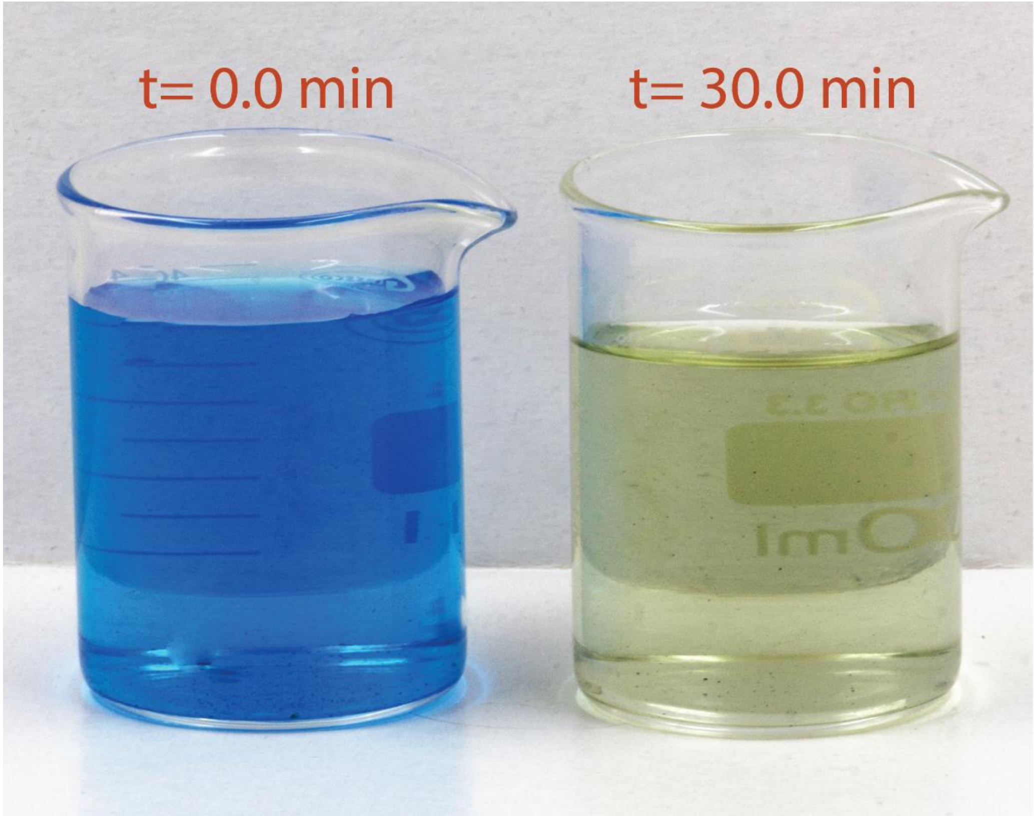

Figure S2. Optical image for adsorption of MB onto rGO at T = 298 K. The MB-rGO suspension was centrifugated at 3000 rpm for 5 min after 30 min of contact time.

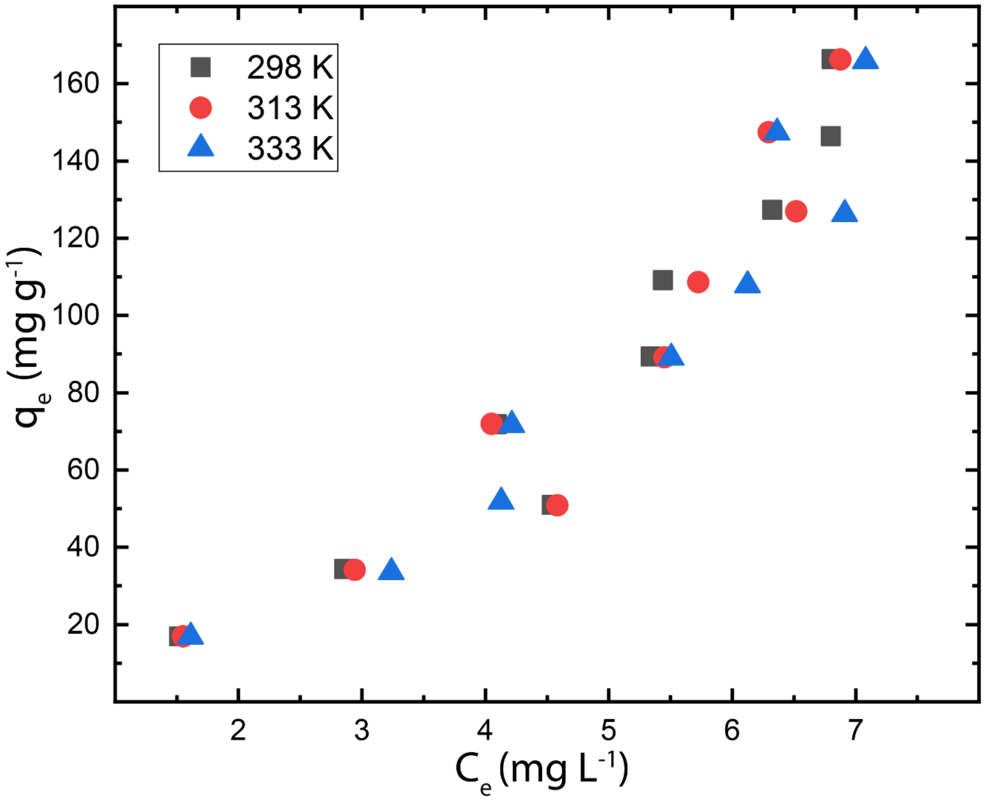

Figure S3. Experimental adsorption isotherms for the removal of MB at different temperatures.
